# Response Surface Optimization of Extraction Conditions for the Active Components with High Acetylcholinesterase Inhibitory Activity and Identification of Key Metabolites from *Acer truncatum* Seed Oil Residue

**DOI:** 10.3390/foods12091751

**Published:** 2023-04-23

**Authors:** Ruonan Meng, Kaixiang Ou, Ling Chen, Yu Jiao, Fangjie Jiang, Ronghui Gu

**Affiliations:** 1 Key Laboratory of Plant Resource Conservation and Germplasm Innovation in Mountainous Region (Ministry of Education), Guizhou University, Guiyang 550025, China; mrn18287438122@163.com; 2 School of Liquor and Food Engineering, Guizhou University, Guiyang 550025, China; scyx0727@163.com (K.O.); cl232589@163.com (L.C.); yu2502837811@163.com (Y.J.); upup55@163.com (F.J.); 3 National & Local Joint Engineering Research Center for the Exploitation of Homology Resources of Medicine and Food, Guizhou University, Guiyang 550025, China

**Keywords:** *Acer truncatum*, seed oil residue, acetylcholinesterase, active components, response surface methodology, UPLC-QTOF-MS, Alzheimer’s disease

## Abstract

The State Council of China has called for the comprehensive development and utilization of *Acer truncatum* resources. However, research on one of its by-products, namely seed oil residue (ASR), from seed oil extraction is seriously insufficient, resulting in a waste of these precious resources. We aimed to optimize the conditions of ultrasound-assisted extraction (UAE) using a response surface methodology to obtain high acetylcholinesterase (AChE) inhibitory components from ASR and to tentatively identify the active metabolites in ASR using non-targeted metabolomics. Based on the results of the independent variables test, the interaction effects of three key extracting variables, including methanol concentration, ultrasonic time, and material-to-liquid ratio, were further investigated using the Box–Behnken design (BBD) to obtain prior active components with high AChE inhibitory activity. UPLC-QTOF-MS combined with a multivariate method was used to analyze the metabolites in ASR and investigate the causes of activity differences. Based on the current study, the optimal conditions for UAE were as follows: methanol concentration of 85.06%, ultrasonic time of 39.1 min, and material-to-liquid ratio of 1.06:10 (g/mL). Under these optimal conditions, the obtained extracts show strong inhibitions against AChE with half maximal inhibitory concentration (IC_50_) values ranging from 0.375 to 0.459 µg/mL according to an Ellman’s method evaluation. Furthermore, 55 metabolites were identified from the ASR extracted using methanol in different concentrations, and 9 biomarkers were subsequently identified as potential compounds responsible for the observed AChE inhibition. The active extracts have potential to be used for the development of functional foods with positive effects on Alzheimer’s disease owing to their high AChE inhibition activity. Altogether, this study provides insights into promoting the comprehensive utilization of *A. truncatum* resources.

## 1. Introduction

*Acer truncatum*, a perennial deciduous arbor from the genus *Acer* (Sapindaceae), is native to China and has been widely cultivated around the world for its ornamental, ecological, edible, and nutritional values [1]. *A. truncatum* seed oil (ATO) has been considered a high-quality woody oil [2], thanks to its abundant unsaturated fatty acids (up to 90%) and its high content of nutritional fatty acids including nervonic acid (5.52%) [3], linoleic acid (37.3%), and oleic acid (25.8%) [4]. Moreover, ATO has also been reported as a healthcare oil with various health benefits, including antioxidant effects, antitumor properties [5], antibacterial effects [6], hypolipidemic properties [7], enhanced brain nerve activity [8], and enhanced memory effects [9]. Therefore, the Chinese government has approved ATO as a new food resource (National Health Commission of the People’s Republic of China www.nhc.gov.cn, accessed on 25 October 2022) and has called for accelerating the comprehensive development and utilization of this woody oil resource (State Council of China www.gov.cn, accessed on 25 October 2022). Recently, *A. truncatum* has been planted on large scale in China, which provides sufficient seeds for producing ATO and related products. However, the *A. truncatum* seed residue (ASR) generated from ATO extraction is increasing rapidly and is often discarded as waste or simply used as fertilizer and feed, resulting in serious waste due to the insufficient utilization of ASR.

In fact, many seed oil residues still contain a variety of bioactive phytochemicals, indicating that the seed residue resource can be further developed and utilized. For example, it was reported that peony seed residue containing resveratrol, monoterpene glycosides, paeoniflorin, flavonoids, and sterols exhibited antioxidant, antibacterial, antitumor, anti-inflammatory, and cognitive-enhancing activities [10,11,12]. *Camellia oleifera* seed meal also contained bioactive components (e.g., flavonoids, polyphenols, and bioactive glycoproteins) and displayed bioactivities activities, such as antibacterial, insecticidal, antioxidant, and anti-inflammatory potentials [13,14]. The active components in grape seed oil residue, including procyanidins, phenolic acids, and polyphenols, showed considerable antioxidant capacity [15,16,17,18].

As for the phytochemistry and bioactivities of ASR, few studies have been reported. Liu et al. [19] found that *A. truncatum* seed had high contents of crude fiber (5.18 ± 0.06%), total sugar (22.58 ± 0.12%), and protein (29.79 ± 0.52%). Bi et al. [20] reported that *A. truncatum* seed contained 18 kinds of amino acids, and the content of essential amino acids was up to 20.3%. These results indicated that *A. truncatum* seed is rich in nutrients. Moreover, bioactive fractions or compounds were also reported. For instance, Zhang et al. [21] identified 12 compounds (mainly polyphenols and flavonoids) from 70% ethanol extract from *A. truncatum* seed for the first time, and Fan et al. [22] identified 13 phenolic compounds with antioxidant capacities in 70% ethanol extracts from *A. truncatum* seed coats. Our previous study characterized 15 compounds from ASR and found that ASR extracted using 80% methanol showed toxicity on A-549, MCF-7, SW-480, and SMMC-7721 cancer lines. In addition, this ASR extract demonstrated a significant inhibitory effect on acetylcholinesterase (AChE) activity with IC_50_ values from ranging 0.12 to 0.23 µg/mL [23]. Therefore, ASR not only contains nutrient and bioactive components, but also exhibits various bioactivities, especially significant AChE inhibition activity. ASR should be considered for in-depth development and utilization, with priority given to the production of AChE inhibitors (AChEIs).

Alzheimer’s disease (AD) is a progressive degenerative disease of the nervous system [24] and is a global public health problem [25]. At present, AChEIs are still the main clinical drug for the treatment of AD. Delaying the hydrolysis of acetylcholine by inhibiting AChE activity in the brain has been considered to be one of the most effective patterns to treat AD [26]. Non-targeted metabolite analysis based on UPLC-QTOF-MS has been used to analyze metabolic pathways or networks and identify the metabolites and their abundance with high accuracy [27,28]. Ultrasound-assisted extraction (UAE) is a highly efficient technique to obtain bioactive extracts from plant materials due to the capabilities of cell rupture and solute diffusion [29]. This method has gained widespread industrial application due to the advantages of low demand for equipment, user-friendliness, and less damage to the ingredients [30].

Although our previous study found that ASR showed a significant inhibitory effect on AChE activity and suggested the presence of metabolites with strong anti-AChE activity, the optimal UAE extraction conditions for large-scale preparation of AChE-inhibited ASR extracts and the associated bioactive compounds in the ASR are still lacking. Therefore, in this work, UAE conditions, such as extraction solvent concentration, extraction time, and material-to-liquid ratio, were optimized using response surface methodology (RSM) and BBD for the extraction of high anti-AChE components from ASR. Furthermore, the non-targeted metabolomics based on UPLC-QTOF-MS were used to analyze metabolites in ASR and characterize the main chemicals responsible for the observed activity. This study could promote the development and utilization of ASR in the direction of its high-value-added applications against AD, such as in the development of functional foods for the prevention and treatment of AD, and therefore contribute to the comprehensive exploitation of *A. truncatum* resources.

## 2. Materials and Methods

### 2.1. Samples and Reagents

The fruits of *Acer truncatum* were collected in Mandougacha, Ganqika Town, Horqin Left Wing Rear Banner, Tongliao City, Inner Mongolia Autonomous Region, China, in 2020 (42°51′ N, 122°14′ E). The voucher specimen (YS-YBF-2020-TL) was confirmed as *Acer truncatum* by Dr. Ronghui Gu from Guizhou University and deposited at the National & Local Joint Engineering Research Center for the Exploitation of Homology Resources of Medicine and Food at Guizhou University, China. The samples were dried in the sun, hulled, ground, and passed through 40 meshes. The powdered samples were then extracted with n-hexane (0.5 g: 10 mL) in an ultrasonic apparatus (KQ3200E, Kunshan Ultrasonic Instrument, Kunshan, China) for 30 min with an interval of 4 h between extractions. The extraction was repeated five times. The supernatant was then removed after standing and stratified, and the solids in the bottom were dried to obtain ASR. The dried ASR samples were stored at −20 °C before analysis.

Acetylcholinesterase was purchased from Sigma-Aldrich (St. Louis, MO, USA). Acetylthiocholine iodide and 5,5′-Dithio bis-(2-nitrobenzoic acid) were purchased from Macklin Biochemical Technology (Shanghai, China). Sodium dodecyl sulfate SDS was obtained from Solarbio Technology (Beijing, China). Dibasic sodium phosphate and sodium dihydrogen phosphate were purchased from Zhiyuan Chemical Reagent (Tianjin, China). Analytical-grade methanol was obtained from Chuandong Chemical (Chongqing, China). Chromatographic-grade acetonitrile, methanol, and ultrapure water were obtained from Merck (Darmstadt, Germany), and formic acid was obtained from Anpel Technology (Shanghai, China). 

### 2.2. Ultrasound-Assisted Extraction Process

In a 15 mL test tube, ASR samples of varying weights were mixed with 10 mL of different concentrations of methanol solutions, followed by shaking (100 rpm) for 5 min and ultrasonic extraction (KQ3200E, Kunshan Ultrasonic Instrument, Kunshan, China) at different times. The samples were then centrifuged (H1850, Xiangyi Centrifuge Instrument, Changsha, China) at 3500 rpm for 5 min, and the extraction process was repeated twice. The combined supernatant was dried at 50 °C and stored at −20 °C until use.

### 2.3. In Vitro AChE Inhibitory Activity Assays

An AChE inhibitory activity assay was performed in a 96-well plate according to a modified Ellman’s method [31]. Briefly, ASR was dissolved in phosphate-buffered saline (PBS) solution (0.1 M, pH 8.0) and then diluted with PBS to obtain sample solutions with concentrations of 100, 50, 25, 10, and 2.5 µg/mL, respectively. Subsequently, 100 µL of PBS solution, 20 µL of AChE solution (0.2 U/mL), and 20 µL of sample solution were added to each well and mixed thoroughly. The plates were incubated at 37 °C for 10 min and further incubated at 37 °C for 20 min after 20 µL of ATCI (2 mM, PBS as the solvent) was added. Finally, 20 µL of sodium dodecyl sulfate (0.1 M, SDS) and DTNB (2 mM) were added into each well as terminate and chromogenic reagents, respectively. Thus, the final sample concentration of each sample in the well changed to 10, 5, 2.5, 1, and 0.25 µg/mL, respectively. The absorbance value was measured at 405 nm using a microplate reader (Thermo Fisher Scientific, Waltham, MA, USA). The blank control was conducted with PBS instead of the samples. The background control used the same volume of PBS to replace the sample and AChE solution. The inhibitory rate (%) was calculated according to the following equation:

Inhibitory rate (%) = [A_0_ − (A_2_ − A_1_)]/A_0_ × 100,

where A_0_ is the absorbance of the blank control group, A_1_ is the absorbance of the background control group, and A_2_ is the absorbance of the sample group.

The inhibitory rates were expressed as the average of three repeated experiments and the standard deviation. The half maximal inhibitory concentration (IC_50_) value of enzyme activity was calculated with non-linear regression using SPSS Statistics version 26.0 (IBM SPSS Inc., Chicago, IL, USA).

### 2.4. Optimization of Extraction Process of Active Components on AChE Inhibition from ASR

#### 2.4.1. Ultrasound-Assisted Extraction Independent Variables Assay

To optimize the extraction process, the effects of three key variables on IC_50_ were investigated, including methanol concentration (20, 40, 60, 80, and 95%), ultrasonic time (10, 22, 30, 40, and 50 min), and material-to-liquid ratio (0.425:10, 0.625:10, 0.825:10, 1.025:10, and 1.225:10 g/mL).

#### 2.4.2. Response Surface Methodology Design

RSM was used to evaluate the influences of independent variables, including methanol concentration (X_1_, %), ultrasonic time (X_2_, min), and material-to-liquid ratio (X_3_, g/mL), on the responses of IC_50_ (Y, µg/mL). The Box–Behnken design (BBD) was selected in the response surface test design, and the influence of unexplained variability in the response was minimized via randomized experiments [32]. The variables were evaluated at three levels (1, 0, and −1) containing 17 runs and 5 center points. The level determinations of three variables were evaluated through single-factor analysis (Table 1). The second-order polynomial equation for predicting the optimum parameter in RSM was as follows:

Y = A_0_ + A_1_X_1_ + A_2_X_2_ + A_3_X_3_ + A_12_X_1_X_2_ + A_13_X_1_X_3_ + A_23_X_2_X_3_ + A_11_X_1_^2^ + A_22_X_2_^2^ + A_33_X_3_^2^.

A_0_ represents intercept; A_1_, A_2_, and A_3_ are linear coefficient terms; A_12_, A_13_, and A_23_ are interaction regression coefficient terms; and A_11_, A_22_, and A_33_ are quadratic coefficient terms. Analysis of variance (ANOVA) was employed to determine the significance of the data in the model.

### 2.5. UPLC-QTOF-MS Analysis of ASR Compositions

The sample (10 mg) was added to 120 µL of 75% methanol-water and vortex dissolved. The supernatant was used for UPLC-QTOF-MS analysis, after being centrifuged at 17,000× *g*. An amount of 75% methanol-water was used as a blank sample. Quality control (QC) samples were prepared by mixing all sample solutions in equal proportion to analyze the repeatability and stability of the analytical process under the same treatment.

The extracts of ASR were analyzed using the UPLC system (Waters, Milford, MA, USA) equipped with an ACQUITY UPLC HSS T3 column (1.8 µm, 2.1 mm × 100 mm, Waters, Milford, MA, USA). The mobile phase A was 0.1% aqueous formic acid, and mobile phase B was acetonitrile with 0.1% formic acid. The gradient was used with a flow rate of 0.4 mL/min: 0–3 min, 3–13% B; 3–10 min, 13–40% B; 10–21 min, 40–70% B; 21–24 min, 70–97% B; 24–26 min, 97% B; 26–27 min, 97–3% B; and 27–30 min, 3% B. The column temperature was maintained at 40 °C, and the injection volume was 3 μL.

MS1 and MS2 data were collected using a Triple TOF 5600+ mass spectrometer (AB SCIEX, Framingham, MA, USA). In each data collection cycle, the molecular ions with the strongest intensity in MS1 were selected to collect the corresponding MS2 data, and both MS1 and MS2 scan ranges were 50–1200 *m*/*z*. The ESI operation parameters were set as follows: atomization pressure (GS1) of 60 psi, auxiliary pressure of 60 psi, curtain pressure of 35 psi, source temperature of 650 °C, and ion spray voltage of 5000 eV.

### 2.6. Identification of Compounds and Statistical Analysis

The raw data collected through UPLC-QTOF-MS were converted to .*abf* format using an ABF converter (https://www.reifycs.com/AbfConverter/), and the *.abf*-format data were imported into MS-DIAL 4.7 for data processing [33]. Data processing included data collection, peak detection, metabolite identification, adduct-ion merging, and isotope tracking. Information of on retention time, compound molecular weight, molecular formula, peak area, and identification were exported. Confirmation of the putative identifications was performed by checking compound fragments (−10 ppm ≤ mass error ≤ 10 ppm) in Peak View 1.2 (AB SCIEX, Framingham, MA, USA) and previously published reported data.

MetaboAnalyst 5.0 was used for multivariate statistical analysis, including principal component analysis (PCA), partial least squares discriminant analysis (PLS-DA), orthogonal partial least squares discriminant analysis (OPLS-DA), metabolite classification, and enrichment analysis. The differential metabolites in OPLS-DA were screened using variable importance projection (VIP) values and the results of fold change (FC) and t-test from ANOVA, with parameter settings of *p* value < 0.05, VIP ≥ 1, and log_2_ FC ≥ 1 or ≤−1.

## 3. Results and Discussion

### 3.1. Determination of the Range of Independent Variables

**Figure 1:**
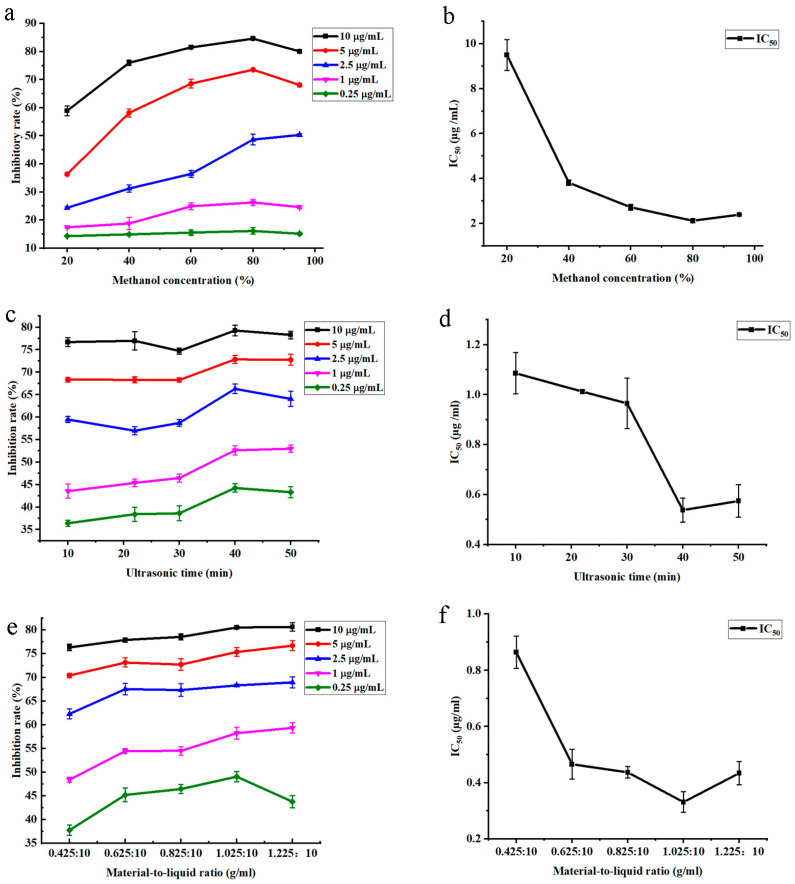
Effects of the methanol concentration (**a**,**b**), ultrasonic time (**c**,**d**), and material-to-liquid ratio (**e**,**f**) on AChE inhibition rates and IC_50_.

The influence of methanol concentration, ultrasonic time, and material-to-liquid ratio on AChE inhibition rate and IC_50_ values were studied first. As shown in Figure 1a, the inhibition rates of AChE increased significantly when the methanol concentration was less than 80% and gradually decreased when the methanol concentration exceeded 80%, except for the concentration of 2.5 µg/mL. When the ASR concentration was 2.5 µg/mL, the inhibition rate of AChE increased with an increase in methanol concentration, but its growth rate slowed down when the methanol concentration exceeded 80%. When the ASR concentration was 10, 5, 1, or 0.25 µg/mL and methanol concentration was 80%, the AChE inhibition rate reached the maximum value, and IC_50_ reached its minimum value (IC_50_ = 2.1216 ± 0.0888 µg/mL, Figure 1b). It has been reported that solvents with low methanol concentrations are more effective in separating water-soluble substances such as polysaccharides, proteins, and pigments. However, these solvents may also lead to the extraction of impurities in the obtained extract [34]. In addition, a previous study also showed that increasing the methanol concentration was conducive to the dissolution of flavonoids [35]. The concentration of 80% methanol may be more suitable for extracting the AChE inhibitory component from ASR due to its polarity, resulting in relatively better activity. From the above results, the methanol concentrations ranging from 65% to 95% were selected to establish the models. 

As shown in Figure 1c, the inhibition rates of AChE fluctuated and improved with the ultrasonic time from 10 min to 40 min, peaked at 40 min, and then slowly declined with increasing ultrasound time. When the ultrasonic time was 40 min, the inhibition rate of AChE reached the maximum value, and the minimum value of IC_50_ was 0.5380 ± 0.0485 µg/mL (Figure 1d). Research has shown that longer ultrasound times results in higher yields of active ingredients. However, prolonged ultrasonic time can lead to an increase in the temperature of the extraction medium, reducing the solvent’s permeability into the cell wall [36]. This can result in the precipitation of impurities, disruption of the structure of small molecules, and degradation or transformation of flavonoids [37]. Therefore, the ultrasound time from 30 min to 50 min was chosen to obtain the models.

As depicted in Figure 1e, with the exception of ASR concentration of 0.25 µg/mL, the inhibition rates of AChE increased with the increase in the material-to-liquid ratio. However, when the material-to-liquid ratio exceeded 1.025:10 (g/mL), the growth rate slowed down. When the material-to-liquid ratio was 1.025:10 (g/mL), the IC_50_ was the minimum value (IC_50_ = 0.3310 ± 0.0368 µg/mL, Figure 1f). Studies have shown that the driving force of mass transfer in ultrasonic extraction is the concentration gradient between solid and solvent [38]. Generally, the rate of active substance extraction from the solid matrix to the solvent increases with the concentration gradient. However, this effect becomes weaker when the ratio of solvent to solid is too high [39]. The contact area between ASR and solvent decreases as the material-to-liquid ratio increases, which can result in some active materials not being effectively dissolved or the dissolution rate slowing down [40]. In this study, a material-to-liquid ratio range of 0.825:10 to 1.225:10 (g/mL) was selected for model building, taking into account the need for economic and efficient extraction.

### 3.2. RSM Optimization of Extraction Condition for the Active Components

#### 3.2.1. Box–Behnken Design and Model Fitting

The BBD experiment design and results are presented in Table 2, and the second-order polynomial equation showing the effect of methanol concentration (X_1_, %), ultrasonic time (X_2_, min), and material-to-liquid ratio (X_3_, g/mL) on IC_50_ (Y, µg/mL) is expressed as follows:

Y = 0.3511 − 0.3373 X_1_ + 0.0164 X_2_ − 0.0229 X_3_ − 0.0111 X_1_X_2_ + 0.0435 X_1_X_3_ + 0.0385 X_2_X_3_ + 0.4873 X_1_^2^ + 0.1070 X_2_^2^ + 0.0349 X_3_^2^.

**Figure 2:**
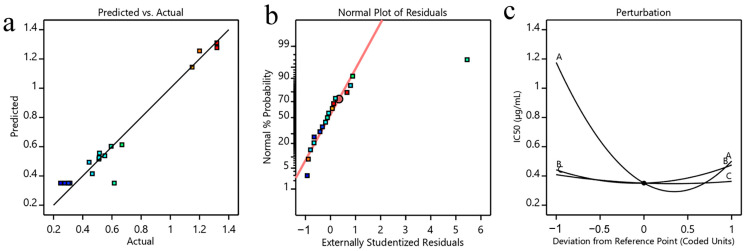
Data diagnosis plot for the models: (**a**) predicted vs. actual plot, (**b**) normal plot of residuals, and (**c**) perturbation plot. A: methanol concentration, B: ultrasonic time, and C: material-to-liquid ratio. The different colors represent the dispersion of the discrete random variables.

To test the validity and predictability of the model, the results were analyzed using ANOVA (Table 3). The *p*-value and *F*-value were used to estimate the statistical significance of the model. The *p*-value < 0.05 implies that the model is significant and the *p*-value < 0.01 implies that the model is extremely significant. It was observed that the model was significant (*p* < 0.05), and the lack of fit was insignificant (*p* > 0.05). These results indicated that the established model could be used to predict the IC_50_ from the extraction conditions (methanol concentration, ultrasonic time, and material-to-liquid ratio). The correlation coefficient value (R^2^) was 0.9505, indicating that 95.05% of the IC_50_ value could be explained with the regression model. However, R^2^ may not be accurate when there are many variables that continue to rise. Thus, the adjusted determination coefficient value (R_Adj_^2^) was often used to replace R^2^ and further validated the significance between independent variables and responses [41]. The predicted values obtained from the second-order polynomial equations were close to the experiment values and scattered around the theoretical line, as illustrated in Figure 2a. Figure 2b is the normal plot of the residual, and the spot approximately along the straight line represents the acceptable reproducibility of the method. These results indicated that the relationship between the test and predicted values was reliable and accurate.

Moreover, it was found that in addition to linear (X_1_) and quadratic (X_1_^2^) coefficients, the other variables did not show significant effects on the IC_50_ values (Table 3). Figure 2c depicts the perturbation plot of the IC_50_; the steep curvature with the methanol concentration demonstrated that the IC_50_ is rapidly responsive to this factor, while the relatively flat line of the ultrasonic time and material-to-liquid ratio indicates their minimal effect on the IC_50_. In summary, the factors affecting IC_50_ in descending order of importance were X1 followed by X3 and X2.

#### 3.2.2. Analysis of the Variable Interaction

**Figure 3:**
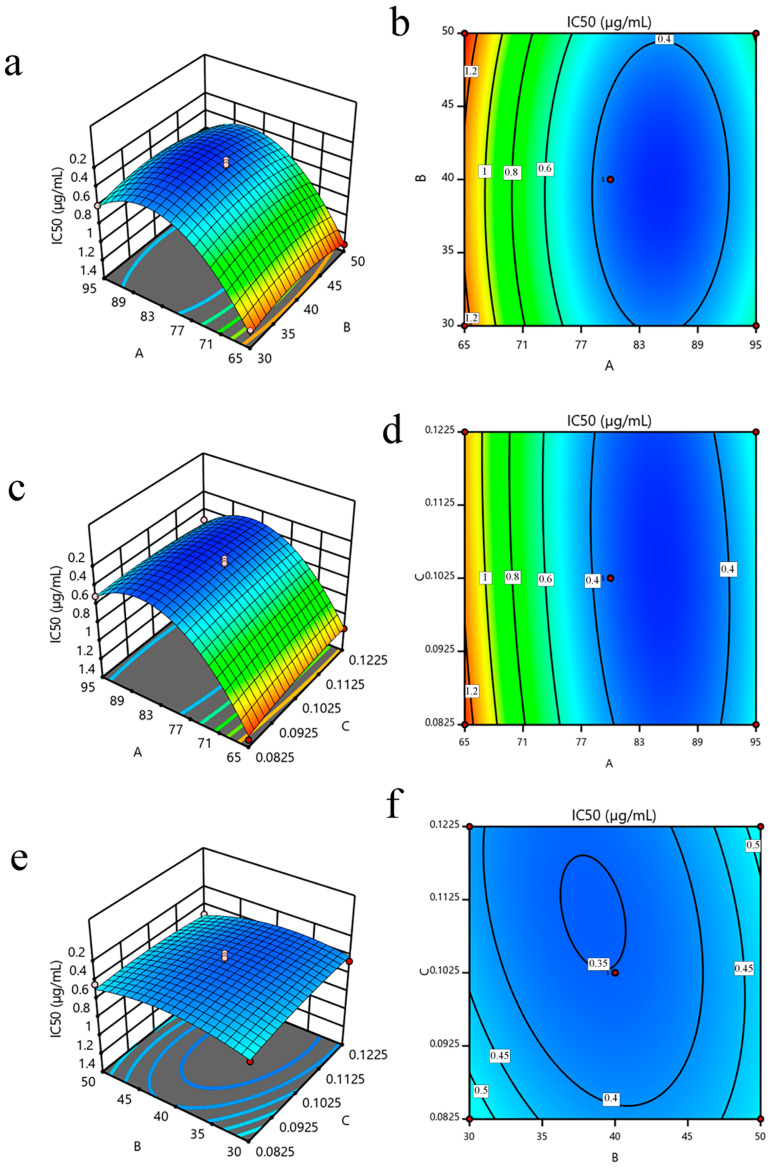
Response surface and contour plots: (**a**,**b**) methanol concentration vs. ultrasonic time, (**c**,**d**) methanol concentration vs. material-to-liquid ratio, and (**e**,**f**) ultrasonic time vs. material-to-liquid ratio. A: methanol concentration, B: ultrasonic time, and C: material-to-liquid ratio.

The three-dimensional (3D) response surface and contour plots were generated to facilitate the visualization of the significant variables and explore the interaction of each factor, as illustrated in Figure 3. The IC_50_ decreased as the interaction between methanol concentration and ultrasonic time increased up to an optimum point, after which it slowly increased (Figure 3a,b). Notably, the negative model term (−0.0111 X_1_X_2_) reveals a confrontational behavior between the variables, indicating that the IC_50_ may decrease with higher methanol concentrations and longer ultrasonic times. 

Figure 3c,d represent the impact of the interaction between methanol concentration and material-to-liquid ratio on the IC_50_. Compared to the interaction between methanol concentration and ultrasonic time, this interaction was more prominent. Interestingly, the positive model term (+0.0435 X_1_X_3_) indicates a synergistic effect, confirming that decreasing both factors to their minimum values leads to a reduction in the IC_50_.

Figure 3e,f elucidate the response surface and contour plots of the interaction of ultrasonic time and material-to-liquid ratio. The positive model term (+0.0385 X_2_X_3_) promulgates a synergistic effect between the variables. It was noticed that the interaction between various variables was not significant (*p* > 0.05, Table 3). 

#### 3.2.3. Optimization and Validation of the Model

According to the Design Expert 11.1.0 software (Stat-Ease Inc., Minneapolis, MN, USA), the optimal extraction conditions of the active components, in terms of AChE inhibition in ASR, were as follows: methanol concentration of 85.06%, ultrasonic time of 39.1 min, and material-to-liquid ratio of 1.06:10 (g/mL), whereby the predicted IC_50_ value (Y) was 0.292 µg/mL. Under these optimum conditions, the observed value in the verification test ranged from 0.375 to 0.459 µg/mL, which was slightly different compared to the predicted one, suggesting that the established optimized conditions were reliable and that the regression model was suitable for extracting the active ingredients from ASR. The IC_50_ measured value was different from that of our previous study, which may be due to the data deviation caused by various factors, such as different years of material harvesting, different test operators, and environment conditions. Additionally, the activity of AChE can be easily influenced by environmental factors. However, the deviation is small and within acceptable limits. Overall, ASR shows a strong AChE inhibitory activity. 

### 3.3. Non-Targeted Metabolite Analysis of ASR

**Figure 4:**
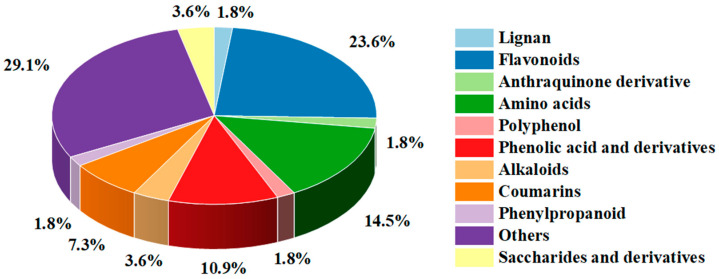
The types and proportion of tentatively identified metabolites.

To better screen components with high anti-AChE activity from ASR, we performed non-targeted UPLC-QTOF-MS-based metabolite analysis of methanol extracts with different concentrations (20%, M1; 40%, M2; 60%, M3; 80%, and M4; 95%, M5) because the methanol concentration is the most influential factor for IC_50_ based on the results of independent variable analysis. The base peak ion (BPI) chromatograms under positive and negative ion modes are shown in Figures S1–S5. Figures S6–S8 show the MS2 spectrum and fragmentation pathway of *L*-Epicatechin, quercetin, and luteolin. In total, 55 metabolites were tentatively identified (Table 4, Figure 4), including 13 flavonoids, 8 amino acids, 6 phenolic acids and derivatives, 4 coumarins, 2 saccharides, and derivatives as well as 2 alkaloids, 1 lignan, 1 polyphenol, 1 phenylpropanoid, 1 anthraquinone derivative, and 16 others.

### 3.4. Multivariate Statistical Analysis

**Figure 5:**
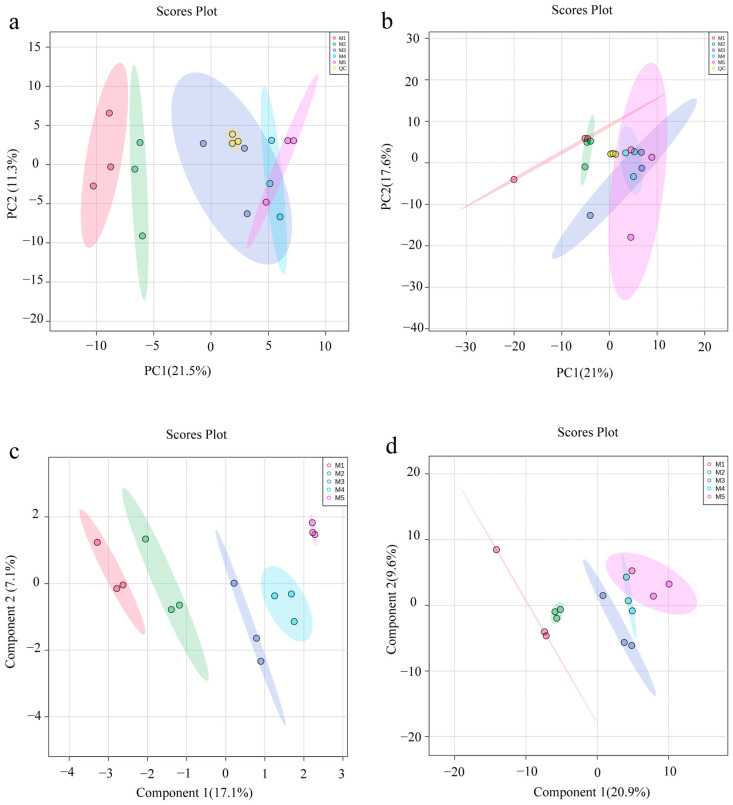

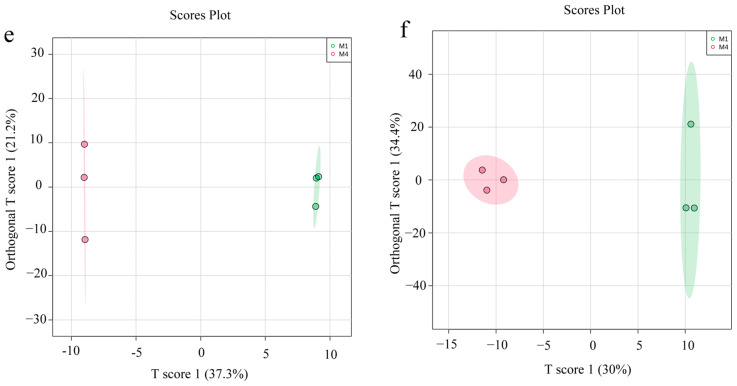
Multivariate analysis score plots ((**a**): ESI^+^, PCA; (**b**): ESI^−^, PCA; (**c**): ESI^+^, PLS-DA; (**d**): ESI^−^, PLS-DA; (**e**): ESI^+^, OPLS-DA; (**f**): ESI^−^, OPLS-DA).

MS-DIAL software and public database (MS/MS-Public-Pos/Neg 17) were used for data processing, resulting in the characterization of 3486 mass features. Among these, 1598 were found using positive ion mode and 1888 using negative ion mode. These features were exported in the format of *.csv* for the first feature screening to eliminate uncertain and duplicate metabolite information. MetaboAnalyst omics online platform was employed for multivariate statistical analysis based on these 3486 features from ASR extraction using different methanol concentrations (Figure 5). The results show that the QC group overlapped together in PCA, which indicates that the systems were stable during data acquisition. In addition, the PLS-DA model was presented with a satisfactory discriminating ability to divide the five groups in positive ion mode (R^2^Y = 0.9427, Q^2^ = 0.6907), but the difference in negative ion mode is not very obvious (R^2^Y = 0.9919, Q^2^ = 0.7146), especially regarding M4 and M5. Furthermore, the OPLS-DA method was used to further explore the difference between the most active group (M4) and the least active group (M1).

### 3.5. Screening of Differential Metabolites

**Figure 6:**
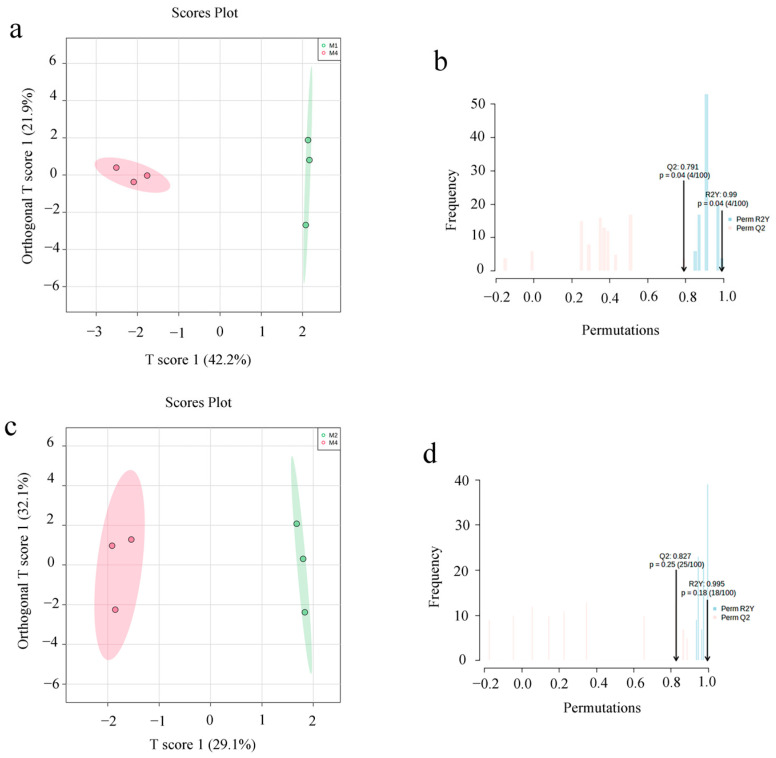

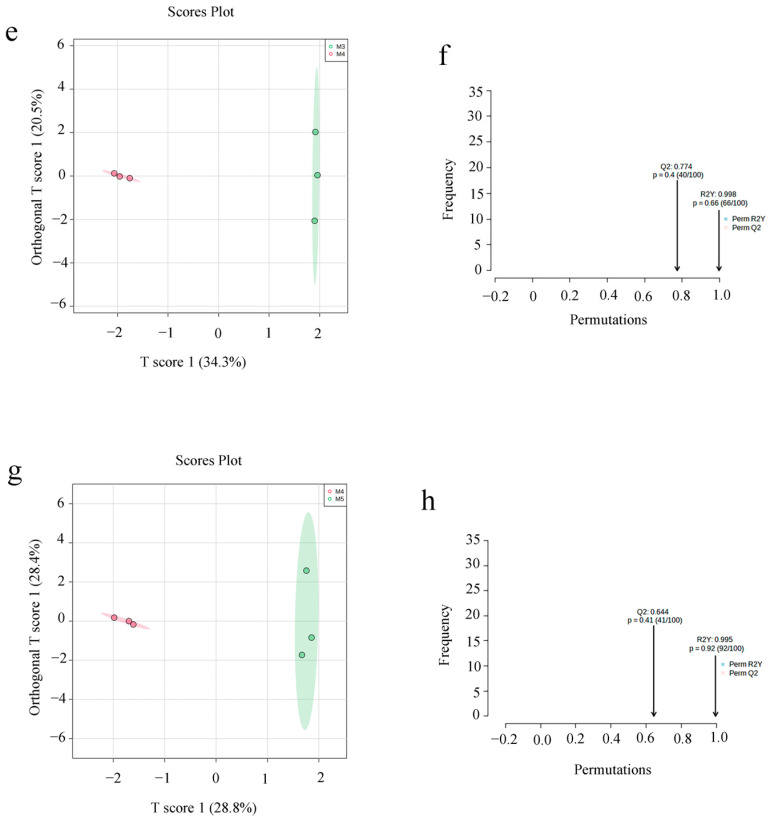
OPLS-DA score plots along with their corresponding validation models of 100 random permutation tests ((**a**,**b**): M4 vs. M1; (**c**,**d**): M4 vs. M2; (**e**,**f**): M4 vs. M3; (**g**,**h**): M4 vs. M5).

**Figure 7:**
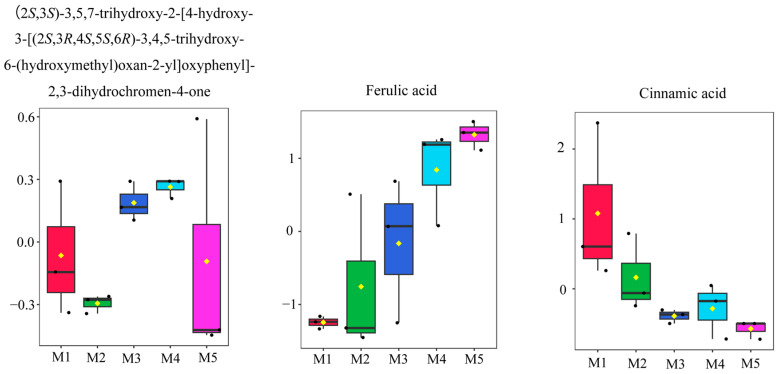

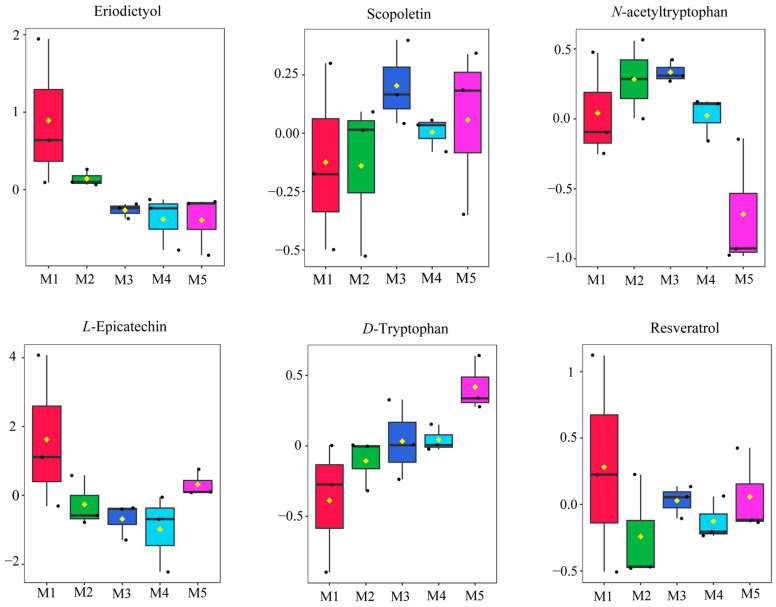
Box plots of the normalized peak intensities of differential compounds from methanol extracts of ASR with different methanol concentrations.

We employed OPLS-DA to evaluate the difference between the 55 metabolites in extracts of ASR with different methanol concentrations, and the results indicated that M4 (the highly active extract) and other groups (the weakly active extract) can be separated (Figure 6). Next, we performed VIP and FC analyses to find the differential metabolites between groups, and a permutation test was applied to validate the OPLS-DA model (Figure 6). R^2^Y and Q^2^ were close to 1, and Q^2^ was higher than 0.5, indicating that the model was stable and reliable. Through the above screening methods, 9 differential metabolites between the two methanol extracts of ASR were tentatively identified (Table 5). Figure 7 shows the normalized peak intensity box plot of the different compounds. Each point on the graph represents a sample, and the content difference of the compounds among the groups can be found distinctly.

Except for (2*S*,3*S*)-3,5,7-trihydroxy-2-[4-hydroxy-3-[(2*S*,3*R*,4*S*,5*S*,6*R*)-3,4,5-trihydroxy-6-(hydroxymethyl)oxan-2-yl]oxyphenyl]-2,3-dihydrochromen-4-one and *D*-Tryptophan, the other seven discriminative metabolites have been reported to display inhibitory effects on AChE or anti-AD. Studies have shown that ferulic acid was the most common active component for the inhibition of Aβ aggregation, and the protection of neurons from oxidative damage, but its interaction with AChE was not significant (<20% inhibition of AChE at 20 mM) [54]. Rollinger et al. separated scopoletin from the roots of *Scopolia carniolica* and determined its IC_50_ value of inhibition AChE activity as 168.6 µM [55]. At a concentration of 50 µg/mL, *N*-acetyltryptophan exhibited a significant AChE inhibition rate of 64.90 ± 1.61%. Therefore, it is considered a promising compound for the treatment of AD [56]. Resveratrol has been proven to protect neuronal cells with its antioxidant activity, improve the memory function of patients with dementia, and reverse AChE activity [57]. The study confirmed that when the concentration of *L*-Epicatechin was 5 mg/mL, the inhibition rate of AChE was 13.48% [58]. Cinnamic acid derivatives showed a good inhibition effect of Aβ (1–42) aggregation and good neuroprotection on PC12 cells against amyloid-induced cell toxicity, indicating that they were promising for further development as lead compounds in the treatment of AD [59]. Eriodictyol can alleviate LPS-induced neuroinflammation, amyloidogenesis, and memory impairment, and has been fully proven to possess excellent anti-inflammatory, antioxidant, and anticancer biological activities [60]. Therefore, we proposed that the differential metabolites screened by OPLS-DA could be the active compounds in the ASR extracts with high AChE inhibitory activity.

## 4. Conclusions

The experimental results indicated that the optimized conditions for UAE were effective in extracting AChE inhibitory components from ASR. The extracts with the IC_50_ values ranging from 0.375 to 0.459 µg/mL were obtained using a methanol concentration of 85.06%, ultrasonic time of 39.1 min, and material-to-liquid ratio of 1.06:10 (g/mL). Furthermore, 55 metabolites were identified from the ASR extracted using different methanol concentrations. Among them, resveratrol, riodictyol, scopoletin, ferulic acid, cinnamic acid, *N*-acetyltryptophan, L-Epicatechin, D-Tryptophan, and (2*S*,3*S*)-3,5,7-trihydroxy-2-[4-hydroxy-3-[(2*S*,3*R*,4*S*,5*S*,6*R*)-3,4,5-trihydroxy-6-(hydroxymethyl)oxan-2-yl]oxyphenyl]-2,3-dihydrochromen-4-one are potential biomarkers. The extracts from ASR obtained through UAE could be used for the development of functional foods with positive effects on Alzheimer’s disease.

## Figures and Tables

**Table 1:** Independent variables and levels used in Box–Behnken design (BBD).

Independent Variables	Levels		
	−1	0	1
X_1_: methanol concentration (%)	65	80	95
X_2_: ultrasonic time (min)	30	40	50
X_3_: material-to-liquid ratio (g/mL)	0.825:10	1.025:10	1.225:10

**Table 2:** The Box–Behnken design and results.

Run	Methanol Concentration (X_1_, %)	Ultrasonic Time (X_2_, min)	Material-to-Liquid Ratio (X_3_, g/mL)	IC_50_ (Y, µg/mL)
1	80	30	0.825:10	0.5510 ± 0.0560
2	80	40	1.025:10	0.3130 ± 0.0810
3	95	40	0.825:10	0.5105 ± 0.0535
4	80	40	1.025:10	0.2480 ± 0.0210
5	95	50	1.025:10	0.6685 ± 0.0595
6	80	50	0.825:10	0.4435 ± 0.0055
7	65	40	1.225:10	1.1490 ± 0.1530
8	65	50	1.025:10	1.3185 ± 0.0035
9	80	40	1.025:10	0.3020 ± 0.0500
10	80	40	1.025:10	0.2765 ± 0.0545
11	95	30	1.025:10	0.5945 ± 0.0255
12	65	40	0.825:10	1.3190 ± 0.0035
13	95	40	1.225:10	0.5145 ± 0.0115
14	80	40	1.025:10	0.6160 ± 0.0560
15	65	30	1.025:10	1.2000 ± 0.0230
16	80	50	1.225:10	0.5120 ± 0.0340
17	80	30	1.225:10	0.4655 ± 0.0525

**Table 3:** ANOVA for the fitted models.

Source	Sum of Squares	df	Mean Square	*F*-Value	*p*-Value	
Model	2.02	9	0.2249	14.93	0.0009	Significant
X_1_-Methanol concentration	0.9102	1	0.9102	60.43	0.0001	
X_2_-Ultrasonic time	0.0022	1	0.0022	0.1435	0.7160	
X_3_-Material-to-liquid ratio	0.0042	1	0.0042	0.2779	0.6143	
X_1_X_2_	0.0005	1	0.0005	0.0329	0.8613	
X_1_X_3_	0.0076	1	0.0076	0.5025	0.5013	
X_2_X_3_	0.0059	1	0.0059	0.3936	0.5503	
X_1_^2^	0.9997	1	0.9997	66.37	<0.0001	
X_2_^2^	0.0482	1	0.0482	3.20	0.1167	
X_3_^2^	0.0051	1	0.0051	0.3403	0.5780	
Residual	0.1054	7	0.0151			
Lack of fit	0.0152	3	0.0051	0.2246	0.8750	Not significant
Pure error	0.0902	4	0.0226			
Cor total	2.13	16				
R^2^ = 0.9505	R^2^_Adj_ = 0.8868	R^2^_pred_ = 0.8196				

Note: Abbreviation: df, degree of freedom.

**Table 4:** Compounds tentatively identified in ASR using UPLC-QTOF-MS analysis.

NO.	Rt (min)	Parent Ions	Error (ppm)	Chemical Formula	MS Fragments	Identifications	References
1	0.532	191.0199 [M−H]^−^	3	C_6_H_8_O_7_	—	Citric acid	[42]
2	0.681	341.1088 [M−H]^−^	−1.3	C_12_H_22_O_11_	323.0971 (C_12_H_19_O_10_) [M−H−H_2_O]^−^, 305.0873 (C_12_H_17_O_9_) [M−H−2H_2_O]^−^, 179.0553 (C_6_H_11_O_6_) [M−C_6_H_11_O_5_]^−^	Trehalose	
3	0.693	119.0353 [M−H]^−^	−6	C_5_H_4_N_4_	—	Purine	[43]
4	0.694	181.0706 [M−H]^−^	3	C_6_H_14_O_6_	163.0609 (C_6_H_11_O_5_) [M−H−H_2_O]^−^, 119.0346 (C_4_H_7_O_4_) [M−2H−C_2_H_5_O_2_]^−^	Sorbitol	
5	0.696	341.1088 [M−H]^−^	−3.9	C_12_H_22_O_11_	—	Sucrose	[44]
6	0.822	343.1228 [M+H]^+^	−2	C_12_H_22_O_11_	163.0604 (C_6_H_11_O_5_) [M−C_6_H_11_O_6_]^+^	2-*O*-alpha-*D*-Mannopyranosyl-*D*-mannopyranose	
7	1.052	146.0921 [M+H]^+^	0.7	C_5_H_11_N_3_O_2_	129.0654 (C_5_H_9_N_2_O_2_) [M−NH_2_]^+^, 128.0818 (C_5_H_10_N_3_O) [M−OH]^+^	4-Guanidinobutyric acid	
8	1.067	124.0391 [M+H]^+^	−4.9	C_6_H_5_NO_2_	106.0292 (C_6_H_4_NO) [M+H−H_2_O]^+^	Nicotinic acid	
9	1.114	206.0813 [M−H]^−^	−3.2	C_11_H_13_NO_3_	164.0716 (C_9_H_10_NO_2_) [M−CO−CH_3_]^−^	*N*-acetyl-*L*-phenylalanine	
10	1.159	130.08 82 [M−H]^−^	8.1	C_6_H_13_NO_2_	—	Leucine	[45]
11	1.161	147.0446 [M−H]^−^	3	C_9_H_8_O_2_	—	Cinnamic acid	[46]
12	1.518	136.0619 [M+H]^+^	7.6	C_5_H_5_N_5_	119.0358 (C_5_H_3_N_4_) [M−NH_2_]^+^	Adenine	
13	1.607	245.0933 [M−H]^−^	3.4	C_13_H_14_N_2_O_3_	116.0504 (C_8_H_6_N) [M−C_5_H_8_NO_3_]^−^, 203.0828 (C_11_H_11_N_2_O_2_) [M−CO−CH_3_]^−^	*N*-acetyltryptophan	
14	1.655	289.0700 [M−H]^−^	−5.2	C_14_H_14_N_2_O_5_	245.0930 (C_13_H_13_N_2_O_3_) [M−COOH]^−^, 203.0822 (C_11_H_11_N_2_O_2_) [M−C_3_H_3_O_3_]^−^	*N*-Malonyltryptophan	
15	1.85	101.0599 [M+H]^+^	9.8	C_5_H_8_O_2_	59.0492 (C_3_H_7_O) [M+H−C_2_H_2_O]^+^, 73.0654 (C_4_H_9_O) [M+H−CO]^+^	5-Valerolactone	
16	2.333	164.0717 [M−H]^−^	6.7	C_9_H_11_NO_2_	147.0449 (C_9_H_7_O_2_) [M−H−NH_3_]^−^	Phenylalanine	
17	5.066	205.0968 [M+H]^+^	−4.2	C_11_H_12_N_2_O_2_	118.0655 (C_8_H_8_N) [M+2H−C_3_H_6_NO_2_]^+^, 132.0804 (C_9_H_10_N) [M+2H-C_2_H_4_NO_2_]^+^	*D*-Tryptophan	
18	5.132	205.0963 [M+H]^+^	−5.6	C_11_H_12_N_2_O_2_	118.0650 (C_8_H_8_N) [M+2H−C_3_H_6_NO_2_]^+^, 132.0814 (C_9_H_10_N) [M+2H−C_2_H_4_NO_2_]^+^	*L*-Tryptophan	
19	5.184	141.0551 [M+H]^+^	−3.7	C_7_H_8_O_3_	109.0294 (C_6_H_5_O_2_) [M−CH_2_OH]^+^, 123.0451(C_7_H_7_O_2_) [M+H−H_2_O]^+^	Gentisyl alcohol	
20	5.202	205.0966 [M+H]^+^	−1.7	C_11_H_12_N_2_O_2_	118.0652 (C_8_H_8_N) [M+2H−C_3_H_6_NO_2_]^+^, 132.0806 (C_9_H_10_N) [M+2H-C_2_H_4_NO_2_]^+^	*DL*-Tryptophan	
21	5.834	146.0583 [M+H]^+^	−1	C_9_H_7_NO	118.0662 (C_8_H_8_N) [M+H−CO]^+^	4-Hydroxyquinoline	
22	6.403	291.0874 [M+H]^+^	2	C_15_H_14_O_6_	39.0378 (C_7_H_7_O_3_) [M+H−C_8_H_8_O_3_]^+^, 123.0475 (C_7_H_7_O_2_) [M+H−C_8_H_8_O_4_]^+^	Catechin	
23	6.486	199.0591 [M+H]^+^	0.5	C_9_H_10_O_5_	—	Syringic acid	[47]
24	6.713	247.1437 [M+H]^+^	−4.1	C_14_H_18_N_2_O_2_	188.0706 (C_11_H_10_NO_2_) [M+H−C_3_H_9_N]^+^, 118.0656 (C_8_H_8_N) [M+2H−C_6_H_12_NO_2_]^+^	Hypaphorine	
25	6.733	183.0302 [M−H]^−^	2.7	C_8_H_8_O_5_	—	Methyl gallate	[48]
26	7.026	169.0490 [M+H]^+^	0.4	C_8_H_8_O_4_	—	Vanillic acid	[46]
27	7.215	369.0826 [M−H]^-^	−1.1	C_16_H_18_O_10_	—	Fraxin	[49]
28	7.221	193.0504 [M+H]^+^	7.1	C_10_H_8_O_4_	65.0550 (C_9_H_9_O_3_) [M+H−CO]^+^, 133.0292 (C_8_H_5_O_2_) [M−CH_3_−CO_2_]^+^	5,7-Dihydroxy-4-methylcoumarin	
29	7.305	209.0435 [M+H]^+^	1.2	C_10_H_8_O_5_	—	Fraxetin	[50]
30	7.405	261.1337 [M−H]^−^	4.7	C_12_H_22_O_6_	187.0972 (C_9_H_15_O_4_) [M−C_3_H_7_O_2_]^−^, 125.0971 (C_8_H_13_O) [M−H−OH−C_4_H_7_O_4_]^−^	9-(2,3-dihydroxypropoxy)-9-oxononanoic acid	
31	7.561	165.0524 [M+H]^+^	−1.3	C_9_H_8_O_3_	—	4-Hydroxycinnamic acid	[46]
32	7.563	123.0445 [M+H]^+^	2	C_7_H_6_O_2_	105.0341 (C_7_H_5_O) [M+H−H_2_O]^+^	Benzoic acid	
33	7.615	289.0708 [M−H]^−^	1.9	C_15_H_14_O_6_	—	*L*-Epicatechin	[22]
34	7.812	357.1329 [M−H]^−^	−6.9	C_20_H_22_O_6_	—	Pinoresinol	[51]
35	7.98	177.0546 [M−H_2_O+H]^+^	0.4	C_10_H_10_O_4_	—	Ferulic acid	[52]
36	8.039	215.1289 [M−H]^−^	−2.7	C_11_H_20_O_4_	153.1277 (C_10_H_17_O) [M−H_2_O−COOH]^−^, 197.1178 (C_11_H_17_O_3_) [M−2H−OH]^−^	Undecanedioic acid	
37	8.18	465.1009 [M−H]^−^	−2	C_21_H_22_O_12_	285.0407 (C_15_H_9_O_6_) [M−2H−C_6_H_11_O_6_]^−^, 303.0518 (C_25_H_11_O_7_) [M−C_6_H_11_O_5_]^−^	(2*S*,3*S*)-3,5,7-trihydroxy-2-[4-hydroxy-3-[(2*S*,3*R*,4*S*,5*S*,6*R*)-3,4,5-trihydroxy-6-(hydroxymethyl)oxan-2-yl]oxyphenyl]-2,3-dihydrochromen-4-one	
38	8.322	273.0756 [M+H]^+^	1.6	C_15_H_12_O_5_	—	Naringenin	[22]
39	8.345	435.1264 [M+H]^+^	−5.5	C_21_H_22_O_10_	—	Naringenin-7-*O*-glucoside	[53]
40	8.38	609.1411 [M−2H]^−^	−2.6	C_27_H_30_O_16_	301.0355 (C_15_H_9_O_7_) [M−H−C_12_H_21_O_9_]^−^	Rutin	
41	8.482	229.0872 [M+H]^+^	1.7	C_14_H_12_O_3_	—	Resveratrol	[46]
42	8.669	191.0326 [M−H]^−^	−1.5	C_10_H_8_O_4_	—	Scopoletin	[46]
43	8.688	303.0503 [M−H]^−^	−4.7	C_15_H_12_O_7_	285.0400 (C_15_H_9_O_6_) [M−H−H_2_O]^−^, 177.0183 (C_9_H_5_O_4_) [M−H−OH−C_6_H_5_O_2_]^−^	Dihydroquercetin	
44	8.706	447.0946 [M−H]^−^	−0.2	C_21_H_20_O_11_	—	Quercitrin	[46]
45	8.716	447.0925 [M−H]^−^	−3.8	C_21_H_20_O_11_	285.0396 (C_15_H_9_O_6_) [M−C_6_H_11_O_5_]^−^	4-(3,4-Dihydroxyphenyl)-5-β-*D*-glucopyranosyloxy-7-hydroxycoumarin	
46	9.269	271.0592 [M−H]^−^	0	C_15_H_12_O_5_	119.0506 (C_8_H_7_O) [M−C_7_H_5_O_4_]^-^, 151.0041 (C_7_H_3_O_4_) [M−2H−C_8_H_7_O]^−^	Naringenin chalcone	
47	9.490	435.1275 [M−H]^−^	−6.4	C_21_H_24_O_10_	273.0763 (C_15_H_13_O_5_) [M−C_6_H_11_O_5_]^−^	Phlorizin	
48	9.518	275.0917 [M+H]^+^	1.8	C_15_H_14_O_5_	107.0505 (C_7_H_7_O) [M−C_8_H_7_O_4_]^+^, 169.0496 (C_8_H_9_O_4_) [M−C_7_H_5_O]^+^	Phloretin	
49	9.633	151.0402 [M−H]^−^	3.5	C_8_H_8_O_3_	—	Vanillin	[46]
50	9.991	243.1587 [M−H]^−^	−0.8	C_13_H_24_O_4_	225.1487 (C_13_H_21_O_3_) [M−H−H_2_O]^−^, 181.1586 (C_12_H_21_O) [M−H−H_2_O−COOH]^−^	Tridecanedioic acid	
51	10.192	271.0614 [M+H]^+^	−2.6	C_15_H_10_O_5_	253.0496 (C_25_H_9_O_4_) [M+H−H_2_O]^+^, 243.0650 (C_14_H_11_O_4_) [M−H−CO]^+^	Emodin	
52	10.368	287.0554 [M−H]^−^	2.4	C_15_H_12_O_6_	—	Eriodictyol	[53]
53	10.507	329.2341 [M−H]^−^	−4.7	C_18_H_34_O_5_	183.1383 (C_11_H_19_O_2_) [M−2H−C_7_H_13_O_3_]^−^	(*Z*)-5,8,11,-trihydroxyoctadec-9-enoic acid	
54	10.544	285.0405 [M−H]^−^	−0.2	C_15_H_10_O_6_	—	Luteolin	[46]
55	10.558	301.0360 [M−H]^−^	−1.3	C_15_H_10_O_7_	—	Quercetin	[22]

**Table 5:** Differential metabolite information for methanol extracts of different concentrations.

No.	Metabolites Name	VIP	log_2_ FC	OPLS-DA Model
1	(2*S*,3*S*)-3,5,7-trihydroxy-2-[4-hydroxy-3-[(2*S*,3*R*,4*S*,5*S*,6*R*)-3,4,5-trihydroxy-6-(hydroxymethyl)oxan-2-yl]oxyphenyl]-2,3-dihydrochromen-4-one	1.5322	2.2256	M4 vs. M1
		1.7243	2.3676	M4 vs. M2
2	Ferulic acid	1.4681	5.8030	M4 vs. M1
		1.2704	1.8072	M4 vs. M2
3	Cinnamic acid	1.3155	−1.2676	M4 vs. M1
4	Eriodictyol	1.0573	−2.0386	M4 vs. M1
		1.2531	−1.6422	M4 vs. M2
5	Scopoletin	1.2856	−1.2213	M4 vs. M3
6	*N*-acetyltryptophan	1.6156	−1.0432	M4 vs. M3
		1.1541	1.1553	M4 vs. M5
7	*L*-Epicatechin	1.4797	−1.3857	M4 vs. M5
8	*D*-Tryptophan	1.5888	−1.1378	M4 vs. M5
9	Resveratrol	1.0606	−1.083	M4 vs. M5

## Data Availability

The data presented in this study are available on request from the corresponding author.

## Supplementary Materials

The following supporting information can be downloaded at https://www.mdpi.com/article/10.3390/foods12091751/s1: Figure S1: BPI of 20% methanol extract (M1) in positive (M1-POS) and negative (M1-NEG) ion modes; Figure S2: BPI of 40% methanol extract (M2) in positive (M2-POS) and negative (M2-NEG) ion modes; Figure S3: BPI of 60% methanol extract (M3) in positive (M3-POS) and negative (M3-NEG) ion modes; Figure S4: BPI of 80% methanol extract (M4) in positive (M4-POS) and negative (M4-NEG) ion modes; Figure S5: BPI of 95% methanol extract (M5) in positive (M5-POS) and negative (M5-NEG) ion modes; Figure S6: The MS2 spectrum and fragmentation pathway of *L*-Epicatechin in negative ion mode; Figure S7: The MS2 spectrum and fragmentation pathway of quercetin in negative ion mode; and Figure S8: The MS2 spectrum and fragmentation pathway of luteolin in negative ion mode.
