# Supplementary material for: Response Surface Optimization of Extraction Conditions for the Active Components with High Acetylcholinesterase Inhibitory Activity and Identification of Key Metabolites from Acer truncatum Seed Oil Residue
Source: Foods. 2023 Apr 23;12(9):1751. doi: 10.3390/foods12091751 (PMC10177952; doi:10.3390/foods12091751)
Supplement: Supplementary file 1 [file foods-12-01751-s001.zip › foods-2314349-supplementary.pdf]

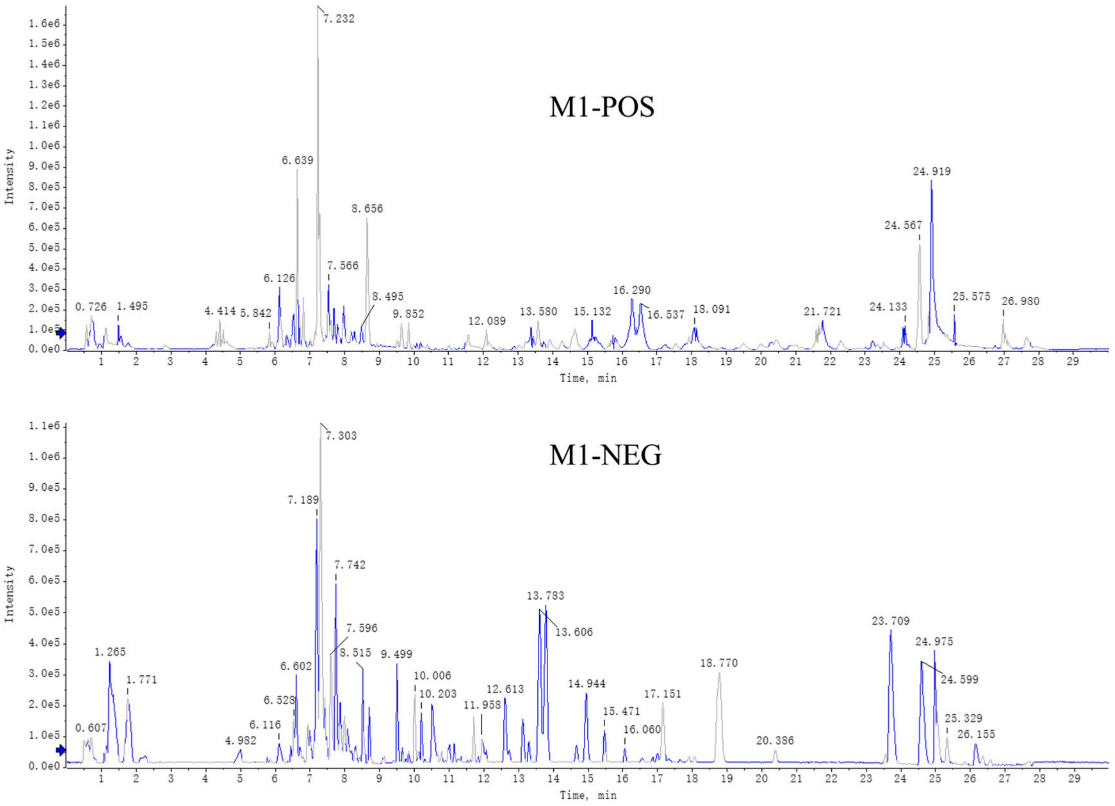

Figure S1. BPI of 20 % methanol extract (M1) in positive (M1-POS) and negative (M1-NEG) ion mode.

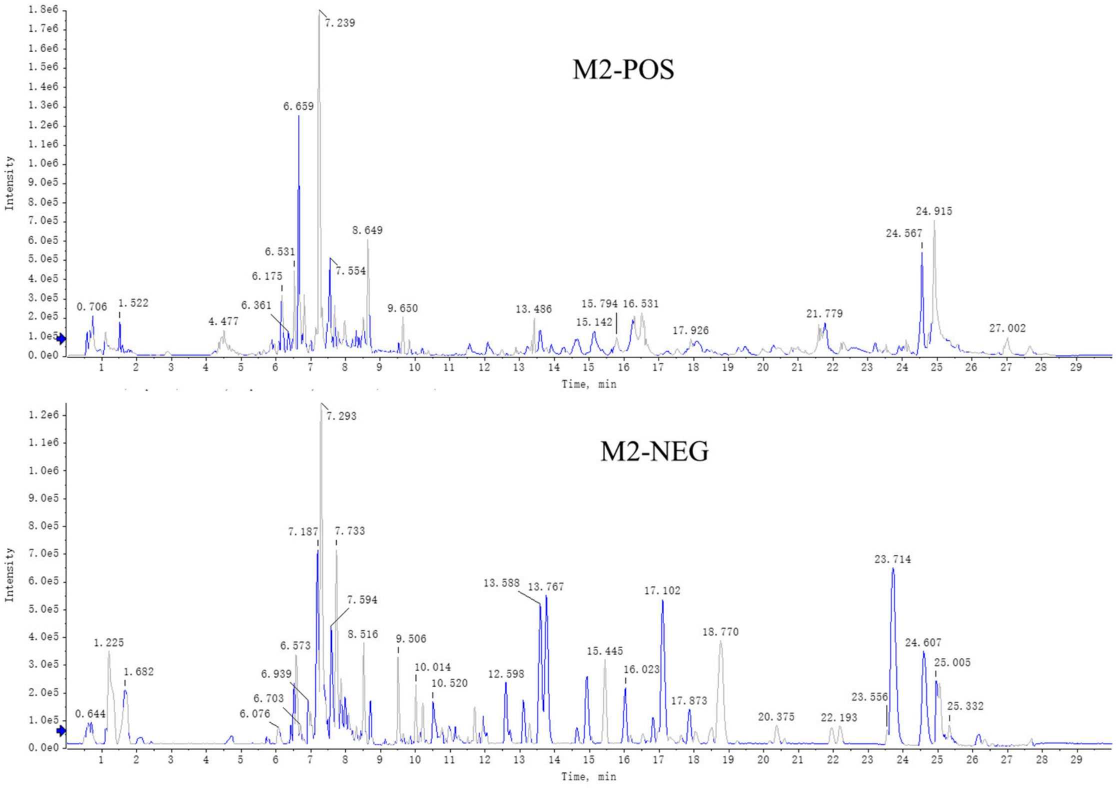

Figure S2. BPI of 40 % methanol extract (M2) in positive (M2-POS) and negative (M2-NEG) ion mode.

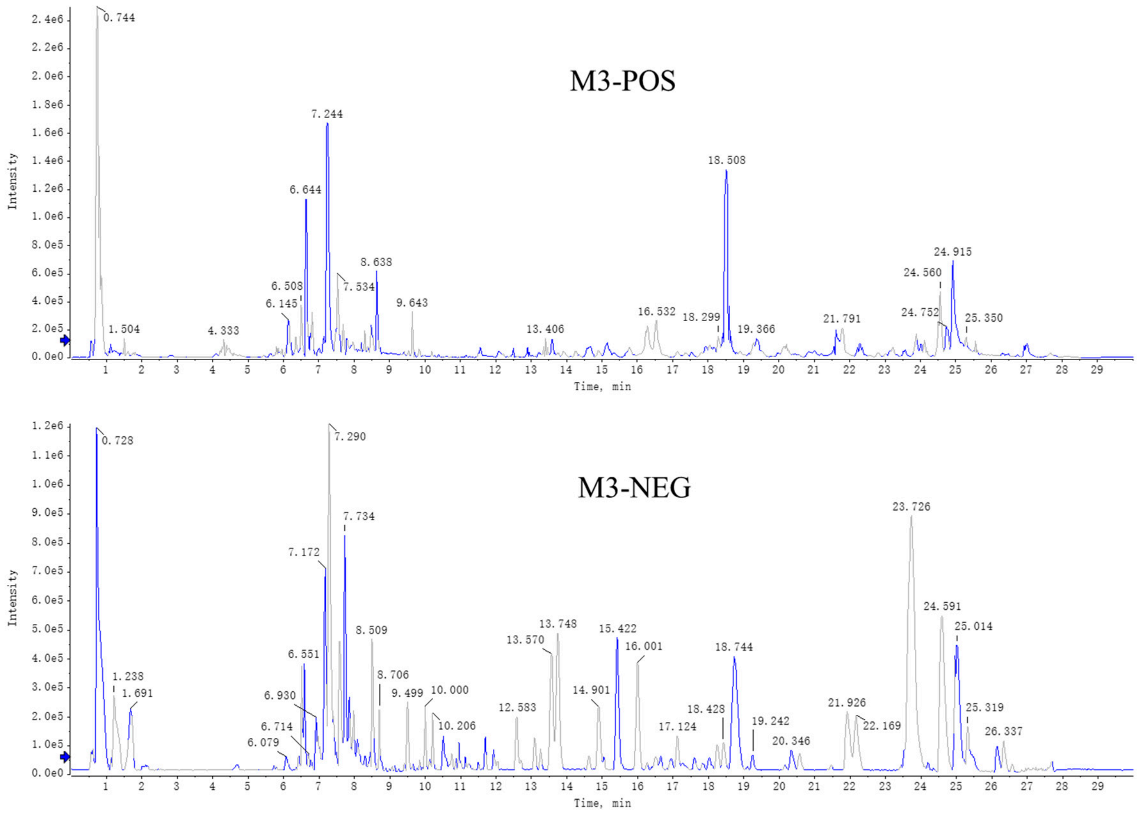

Figure S3. BPI of 60 % methanol extract (M3) in positive (M3-POS) and negative (M3-NEG) ion mode.

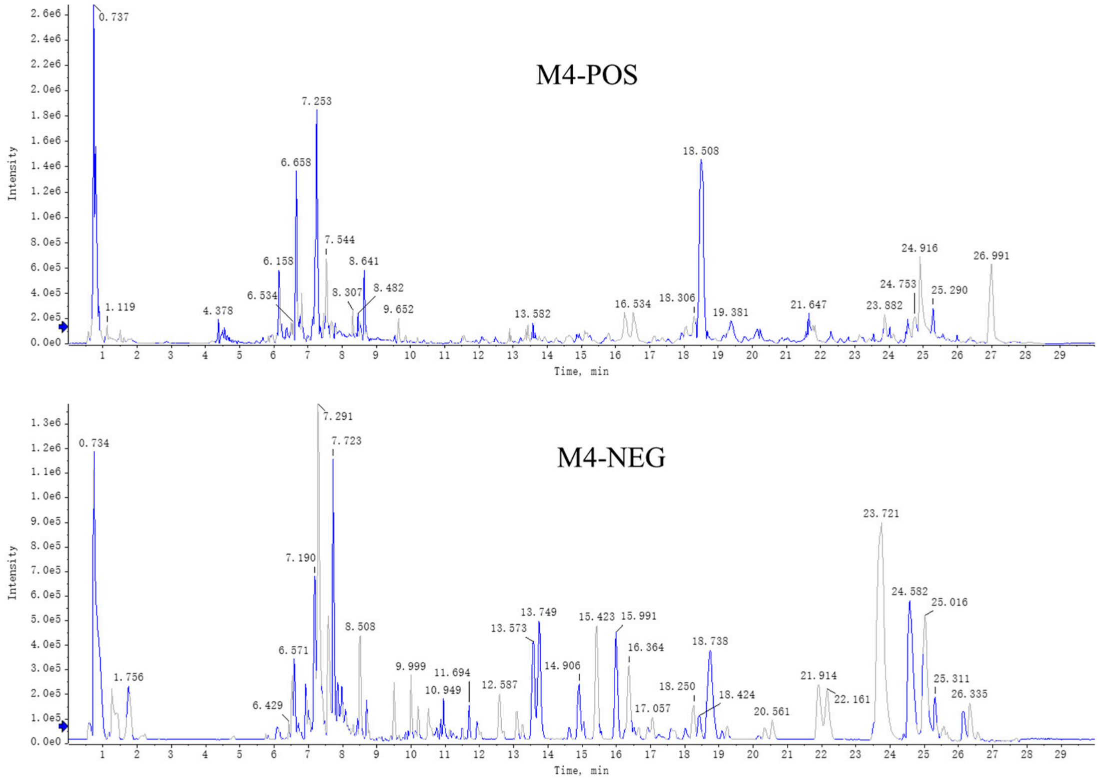

Figure S4. BPI of 80 % methanol extract (M4) in positive (M4-POS) and negative (M4-NEG) ion mode.

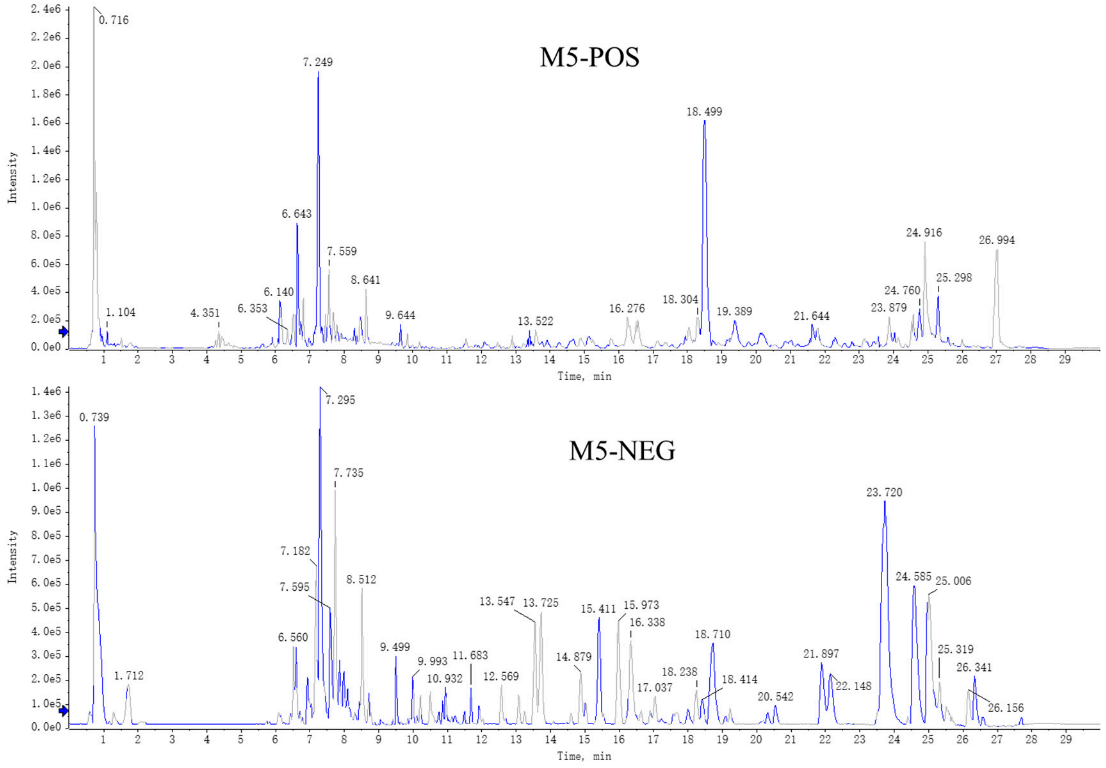

Figure S5. BPI of 95 % methanol extract (M5) in positive (M5-POS) and negative (M5-NEG) ion mode.

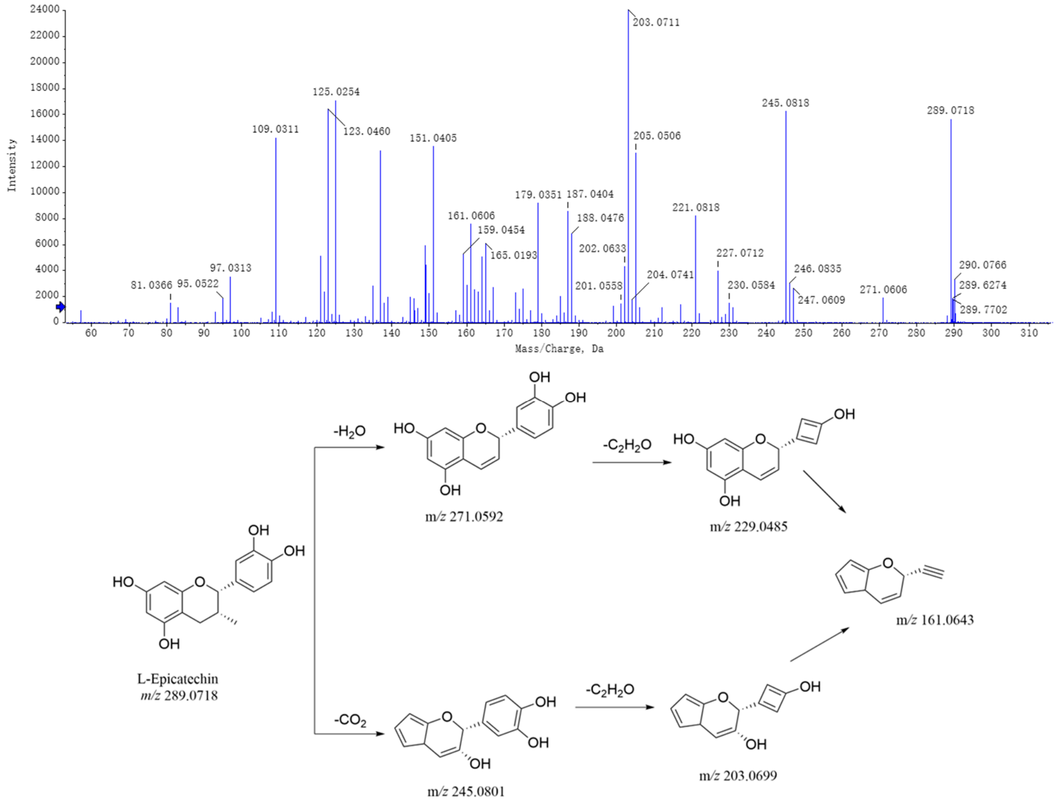

Figure S6. The MS2 spectrum and fragmentation pathway of *L*-Epicatechin in negative ion mode.

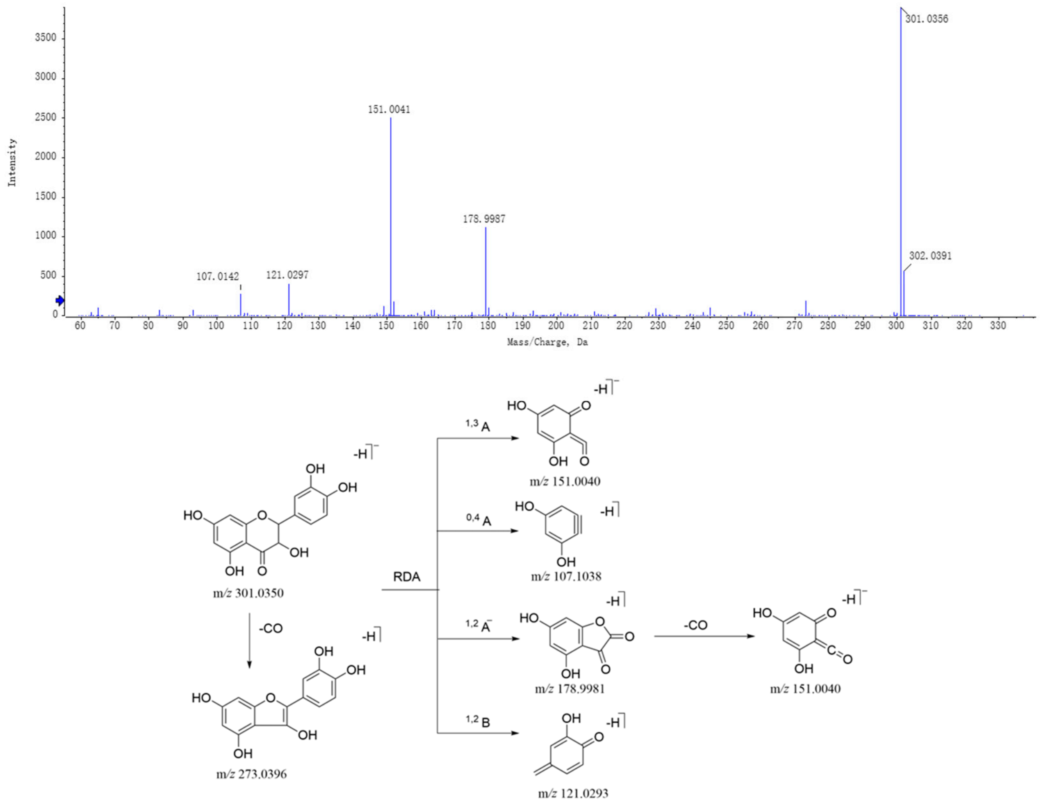

Figure S7. The MS2 spectrum and fragmentation pathway of quercetin in negative ion mode.

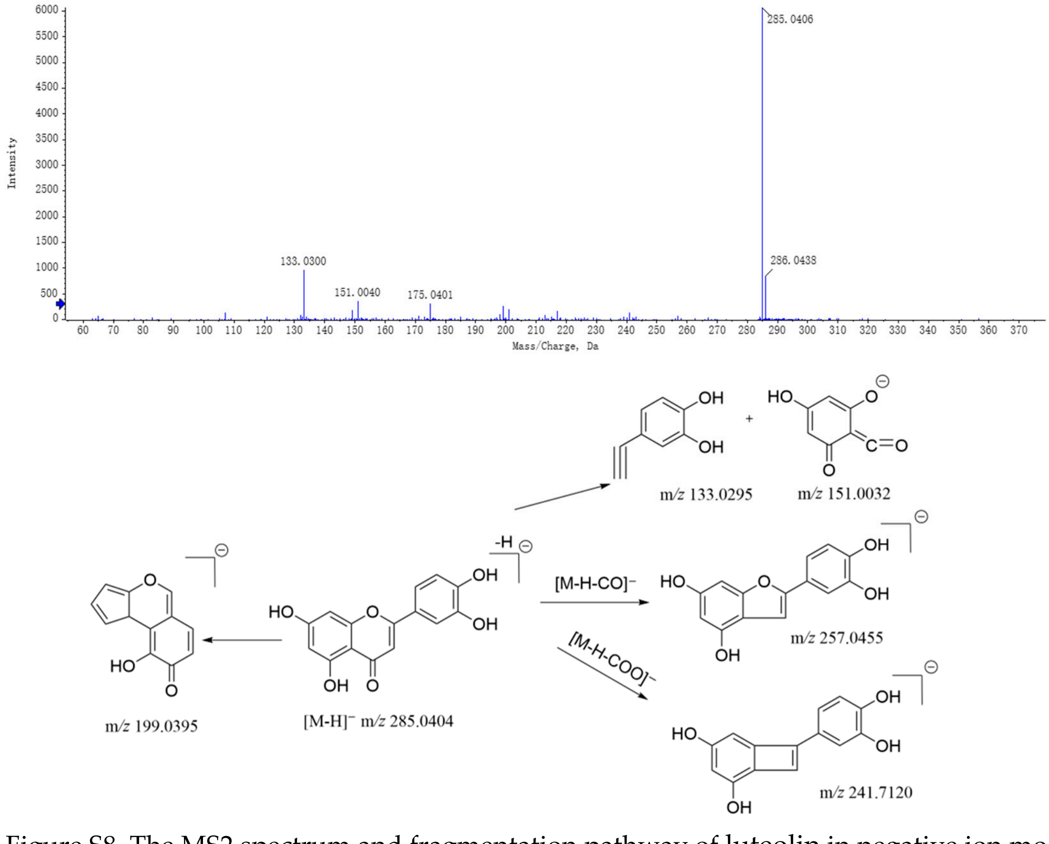

Figure S8. The MS2 spectrum and fragmentation pathway of luteolin in negative ion mode.
